# Radiomics for prediction of survival in lower-grade gliomas—it’s time to move beyond the crystal ball

**DOI:** 10.1007/s00330-020-07603-0

**Published:** 2020-12-19

**Authors:** Daniel Pinto dos Santos, José Maria Garcia Santos

**Affiliations:** 1 Department of Radiology, University Hospital of Cologne, Kerpener Str. 62, 50937 Köln, Germany; 2 Department of Radiology, Hospital General Universitario Morales Meseguer, Murcia, Spain

## Abstract

• *Radiomics might help predict survival of patients with lower-grade gliomas*.

• *Several different models using different radiomics features have been proposed with only little overlap in included features*.

• *Prospective trials and validation studies are needed to establish which models offer clinical benefit and which do not*.

Lower-grade gliomas (LGG, WHO grades II and III) show a wide range of biologic aggressiveness. Accordingly, after the initial surgical resection, there may be a variable need for additional treatment. Decisions on further radiation and/or chemotherapy should be considered on an individual basis [1]. A recent survey in Germany demonstrated high heterogeneity between oncology centers in treatment decisions for LGG [2] and different guidelines have emphasized the idea that much more scientific evidence will be needed for treatment recommendations in the next future [3, 4].

Currently, two options can be considered for LGG when chemotherapy is needed after radiotherapy: PCV (procarbazine, CCNU [lomustine] and vincristine) or temozolomide. PVC is regarded as the more appropriate option in cases of grade II LGG needing treatment beyond surgery. In patients diagnosed with grade III gliomas, chemotherapy is usually contemplated after radiotherapy regardless of the extension of surgical resection or any other risk factor [1]; both PCV and temozolomide would benefit LLG outcomes [1, 3, 4]. Although there are currently no head-to-head comparison between temozolomide and PCV, the former might be more appropriate and is less toxic [1].

While the current study by Wang et al [5] published in this number of European Radiology might not add much evidence as to what therapeutic strategy might be the most appropriate for each individual patient, they aimed at helping clinical decision-making by proposing a radiomics model to predict survival of patients with LGG. They tested their radiomics signature in LGG patients treated with temozolamide to conclude that it has the potential to discriminate patients who may benefit most from this chemotherapy regime.

Certainly, this is no particularly new topic but still their study adds to the idea that one day we might be able to provide individualized treatment decisions based on medical imaging. Recently, two other studies, one by Park et al [6] and one by Choi et al [7], also explored the idea to use radiomics in order to predict survival in patients with LGG. However, if we compare these three studies, one thing becomes very clear: three papers propose five radiomics models and no single radiomic feature is present in all five models. Over the last years, it has become clear, that radiomics research is facing a “reproducibility crisis” that has led to a significant “translation gap” of promising research results into clinical practice [8, 9].

Considering the abovementioned three studies, it is interesting to see that out of 176 features that were included in any of the five models, only five features (“original_glcm_idmn_t1c,” “original_glcm_idmn_t2,” “original_glcm_idn_t2,” “original_firstorder_skewness_t1c,” and “original_glszm_smallareaemphasis_t1c”) were included in three of the five models. These features however, being calculated from contrast-enhanced T1 and T2 sequences respectively, might be subject to relevant test-retest variation [10], whereas many of the features included in the current study by Wang et al [5] were calculated from FLAIR sequences which have been shown to exhibit greater test-retest stability [10]. Even more interestingly, none of the features included in the final model of the study by Wang et al [5] was included in the other studies.

No doubt, radiomic analyses hold the promise to significantly impact patient management by allowing for non-invasive diagnosis and prognosis. However, given the large increase in publications pertaining to this topic and the abovementioned issues, it is difficult to ascertain which model from which publication could indeed be generalizable enough to impact clinical routine. Now might be the time, to move beyond using radiomics to look inside crystal balls and start evaluating already published radiomic models’ predictions and trying to reproduce and understand their results. We have to be able to put promising research results to practical work—and certainly the study by Wang et al [5] is an example of such promising results. This will certainly at some point involve putting such models to the test of prospective studies, in which their clinical value has to be proven in terms of impact on patient outcome. No easy feat of course, but only these next steps will help us move forward.
